# Process controlling iron–manganese regulation of the Southern Ocean biological carbon pump

**DOI:** 10.1098/rsta.2022.0065

**Published:** 2023-06-26

**Authors:** Prima Anugerahanti, Alessandro Tagliabue

**Affiliations:** Department of Earth, University of Liverpool, Ocean, and Ecological Sciences, 4 Brownlow Street, Liverpool L69 3GP, UK

**Keywords:** co-limitation, biogeochemical model, micronutrients

## Abstract

Iron (Fe) is a key limiting nutrient driving the biological carbon pump and is routinely represented in global ocean biogeochemical models. However, in the Southern Ocean, the potential role for other micronutrients has not received the same attention. For example, although manganese (Mn) is essential to photosynthetic oxygen production and combating oxidative stress, it is not included in ocean models and a clear understanding of its interaction with Fe in the region is lacking. This is especially important for the Southern Ocean because both Mn and Fe are strongly depleted. We use a hierarchical modelling approach to explore how the physiological traits associated with Fe and Mn contribute to driving the footprint of micronutrient stress across different phytoplankton functional types (PFTs). We find that PFT responses are driven by physiological traits associated with their physiological requirements and acclimation to environmental conditions. Southern Ocean-specific adaptations to prevailing low Fe, such as large photosynthetic antenna sizes, are of major significance for the regional biological carbon pump. Other traits more strongly linked to Mn, such as dealing with oxidative stress, may become more important under a changing Fe supply regime.

This article is part of a discussion meeting issue ‘Heat and carbon uptake in the Southern Ocean: the state of the art and future priorities’.

## 1. Introduction

Phytoplankton primary production and the export of organic material play a major role in the biological carbon pump [1,2], which controls the atmospheric $p{\text{CO}}_{2}$, and stores dissolved inorganic carbon in the deep ocean [3]. Observational studies report that the concentration of dissolved iron (dFe) in the Southern Ocean is among the lowest found globally [4]. Due to this scarcity, Fe availability is considered a major control on regional phytoplankton growth, net primary production (NPP) and the biological carbon pump [4]. Iron limitation has been studied from the early 1990s, and found in some parts of the Atlantic and Pacific sectors of the Southern Ocean; from the Weddell Sea [5], Drake Passage [6,7] and the Ross Sea [8,9]. In the sixth phase of the Coupled Model Intercomparison Project (CMIP6), earth system models (ESMs) show increasing NPP in the Southern Ocean (with relatively high agreement), while the global trend shows a decline [10–12]. This NPP trend is driven by ocean warming, intensification of the mid-latitude westerly winds and, in the Antarctic zone, disappearing sea ice [10]. Adjustments to Southern Ocean upwelling and increased transport of Fe from subtropical latitudes (due to increased subtropical nitrogen limitation) elevate Fe supply into the Southern Ocean, enhancing regional NPP, the biological carbon pump and ${\text{CO}}_{2}$ uptake [10,13].

Micronutrients, such as Fe, play a key role in underpinning biological contributions to the Southern Ocean carbon cycle as they are used as catalysts in photosynthesis and electron transport [14]. Iron is the most abundant micronutrient in the thylakoid [15], especially in photosystems (PS) I and II (12 atoms in PSI and three atoms in PSII) and is also required during photosynthetic electron transport and several redox reactions [16]. Due to low Fe concentration, Southern Ocean diatoms have less PS to economize Fe, compared with other temperate species [17]. The second most abundant trace metal in the thylakoid is manganese (Mn). In PSII, four Mn atoms are required for water oxidation to form ${\text{O}}_{2}$ [15].

Mn deviates from its usual surface-enriched vertical profile in the Southern Ocean. In the surface waters of the Southern Ocean, Mn concentrations are almost as low as Fe (less than $\;0.4\;\mathrm{nmol}\;l^{-1}$) [18–20], while below 100 m, concentrations increase slightly [21]. This arises because of very low regional dust input and strong upwelling of Mn-depleted waters from the ocean interior, e.g. [7], and is exacerbated by biological uptake [21]. Experimental work using nutrient additions has shown that the phytoplankton community in the Southern Ocean can also respond to addition of volcanic ash (containing both Mn and Fe) [22]. Co-limitation between Mn and Fe has been detected in Drake Passage, both in the western [23] and central part [7] of the passage, and in McMurdo Sound in the Ross Sea [8]. Additionally, the species *Chaetoceros debilis* has been shown to be Mn deficient in the Atlantic sector of the Southern Ocean [19].

ESMs do not consider Mn as a potentially limiting nutrient, and its absence may mean that they underestimate the uncertainty of the response of the Southern Ocean carbon cycle to climate change. Previously, modelling studies have integrated Mn in the coupled global ocean physics-biogeochemical model NEMO-PISCES, but growth limitation was not accounted for [24,25]. Recently, [26] have developed a complete Mn model including Mn limitation and shown that Mn can be an important driver of the response of the Southern Ocean carbon cycle under any regime of changing Fe supply. Traits associated with photophysiology and nutrient acquisition dictate the extent and sensitivity of Mn limitation, but the role of interactions between Fe and Mn and the sensitivity of different phytoplankton functional types remains unknown.

Interactions between Fe and Mn can be driven by how Fe changes Mn demand due to changing growth rates, but also via physiological trade-offs. A key interaction between Fe and Mn occurs in the chloroplast in the physiological response to oxidative stress. Generation of highly reactive intermediates and by-products during photosynthesis can cause oxidative damage, especially to protein synthesis [27]. This occurs in the photosystems when ${\text{O}}_{2}$ is reduced to ${\text{O}}_{2}{\;}^{-}$, which can also lead to production of toxic reactive oxygen species (ROS) and hydroxyl radical (OH⋅) [28]. Notably, the production of ROS increases under Fe stress, because electron flow through photosynthesis is less efficient and ${\text{O}}_{2}$ becomes the electron acceptor [29]. Phytoplankton consume ROS using the antioxidant enzyme superoxide dismutase (SOD), which can require Mn as a cofactor [15,28–30]. Therefore, when internal Fe is depleted, phytoplankton would require more Mn to combat ROS [29,30]. However, at present we have no constraint on how Fe–Mn co-limitation linked to SOD scales up to impact different PFTs at the level of the Southern Ocean region. Moreover, other cofactors can also be used in SOD (e.g. copper, Fe, cadmium and Zn [29–31]) and the extent to which low Mn levels in the Southern Ocean constrain this trade-off is unknown. This knowledge gap also extends to other traits and how they shape phytoplankton growth, NPP and the biological carbon pump. For instance, Mn uptake is inhibited by zinc (Zn, highly abundant in the Southern Ocean [32]), because both trace metals compete for the same transporter [33], which may exacerbate Mn deficiency. Equally, changes in photosynthetic antennae size have been shown to be a critical trait shaping Mn limitation [26], but this also strongly modulates cellular Fe demands [17]. Overall, we lack insight into the interactive impact of Fe and Mn associated with photophysiology and oxidative stress.

Understanding nutrient regulation of growth, NPP and the biological carbon pump requires a consideration of both limiting and deficient nutrients. Limitation refers to the nutrient that limits growth and whose addition alone stimulates additional growth [34]. However, any nutrient that is relatively less abundant in seawater, relative to phytoplankton stoichiometric demands, can be considered to be deficient [34], and can become limiting. Importantly, while Fe and Mn share many common sources, such as wildfires [35], volcanoes [36], dust [22] and melting glaciers [37], differences in the solubility (for dust), bioavailability and effective transport in response to changes in ocean environment due to physical processes, means Mn and Fe can be decoupled. If Fe supply to the Southern Ocean changes, due to external inputs or internal cycling, then unless the Mn supply also increases, Mn limitation may emerge and modulate the response of NPP and the biological carbon pump.

In this study, we focus on Fe–Mn limitation of growth, NPP and the biological carbon pump in a state-of-the-art global ocean model. We examine how a suite of poorly constrained physiological traits, associated with photophysiology, resource acquisition and oxidative stress management, impact Southern Ocean NPP and the biological carbon pump.

## 2. Methods

### (a) Modelling Mn in PISCES-QUOTA

We use the coupled physics and biogeochemical model NEMO-PISCES-QUOTA, with the addition of the micronutrients Mn and Zn. Full details of the PISCES-QUOTA model can be found in [38] and description of general PISCES in [39]. The PISCES-QUOTA model dynamically calculates phytoplankton internal C, N, P, Si, Fe and chlorophyll. Equations concerning the Mn, Zn biogeochemical cycles are fully described in the electronic supplementary material of [24], with detailed equations concerning phytoplankton Mn limited growth and uptakes can be found in [26]. The existing Mn and Zn code for two PFTs is extended for the three PFTs in PISCES-QUOTA (diatoms, nanophytoplankton and picophytoplankton). Below, we describe the calculation of Mn requirement and further additions made in this study. Quota refers to the ratio between internal concentration of nutrients (N, P, Fe, Mn and for diatoms, Si) and the phytoplankton carbon biomass [40]. Similar to how Fe, the Mn requirement for maintaining biomass is computed explicitly for each phytoplankton type. Based on [41], the higher the growth rate and the greater the investment in light harvesting, the more Mn is needed to support phytoplankton growth, defined as the manganese use efficiency (MnUE), which represents the rate that carbon biomass can be produced per catalytic Mn atom

2.1 $${\text{MnUE}}_{i}=\frac{\mu_{i}}{Q_{\text{Mn},\text{Req}}}$$

and

2.2 $$Q_{\text{Mn},\text{req}}=Q_{\text{Mn},\text{min}}+4\times \frac{\text{Chl:C}}{\text{Chl:PSII}}.$$

The subscript i denotes different calculations for the three PFTs in PISCES. The term μ is the specific growth rate and $Q_{\text{Mn},\text{req}}$ is the internal concentration of Mn needed for photosynthesis and basal metabolism. Accordingly, we split the required Mn quota into the Mn required to cover (i) the basal metabolism $Q_{\text{Mn},\text{min}}$, including any requirement for MnSOD, and (ii) the photosynthetic component of phytoplankton, which is described in the second term of equation (2.2). In PISCES-QUOTA, the Chl:C ratio is dynamically calculated, based on the photoacclimation model of [42]. The Chl:PSII ratio represents the photosynthetic antenna size; although phytoplankton can dynamically change its antenna size [17] depending on the internal Fe concentration, a fixed value of 1000 mol mol^−1^ is assumed, with four Mn atoms per PSII. In [26], $Q_{\text{Mn},\text{min}}$ was set to 1 μmol Mn:mol C at a reference growth of $1\;d^{-1}$. Here, we redefined $Q_{\text{Mn},\text{min}}$ to $Q_{\text{MnSOD},\text{min}}$ where we explicitly calculate the need for Mn to activate antioxidant SOD to combat the accumulation of ROS in phytoplankton under Fe stress, generating a new Fe–Mn interaction.

#### (i) Calculating the Mn required to combat ROS

The MnSOD requirement in our model is based on the parameterization of a cellular proteomic model developed for the polar diatom *Fragilariopsis cylindrus* [30]. The underlying philosophy is to represent the inefficiency of electron flow through photosynthesis by making the proportion of electron flow that ‘leaks’ to molecular oxygen $\epsilon_{p}$ a function of the cellular iron limitation. Electron leakage is proportional to photosynthetic energy ($v_{e}$), which increases ROS levels that must then be consumed by MnSOD. Here, electron leakage is dependent on the modelled Fe deficiency ($Q_{\text{def}}^{\text{Fe}}$), which is the ratio of excess Fe quota ($Q_{\text{Fe}}$ minus the iron required for cell maintenance and growth ($Q_{\text{Fe},\text{req}}$)) and the optimum iron quota ($Q_{\text{Opt}}^{\text{Fe}}$). The optimum iron quota refers to the quota when luxury uptake can occur [43]. When the cell is Fe deficient ($Q_{\text{def}}^{\text{Fe}}<1$), $\epsilon_{p}$ is fixed at a maximum value of 30%, and when the cell is Fe replete ($Q_{\text{def}}^{\text{Fe}}>$ 1), $\epsilon_{p}$ is fixed at a lower bound of 5%.

The production rate of ROS (ω) is therefore proportional to $\epsilon_{p}$

2.3 $$\omega =\frac{\epsilon_{p}v_{e}-\epsilon_{a}v_{\text{ROS}}}{\epsilon_{p}v_{e}+\epsilon_{a}v_{\text{ROS}}},$$

where $\epsilon_{a}$ is the efficacy of ROS consumption by MnSOD. In this study, we use the light limited growth rate to approximate $v_{e}$. The rate of ROS consumption $v_{\text{ROS}}$ is then the product of maximum turnover rate of MnSOD ($k_{\text{catROS}}$, MnSOD) and the number of MnSOD copies per cell

2.4 $$v_{\text{ROS}}=k_{\text{catROS}}\times [\text{MnSOD}].$$

Rearranging these three equations, the amount of MnSOD per cell required to balance the ROS production is

2.5 $$\begin{array}{rl} & \frac{\epsilon_{p}v_{e}-\epsilon_{a}v_{\text{ROS}}}{\epsilon_{p}v_{e}+\epsilon_{a}v_{\text{ROS}}}=0, \end{array}$$

2.6 $$\begin{array}{rl} & \epsilon_{p}v_{e}=\epsilon_{a}v_{\text{ROS}} \end{array}$$

2.7 $$\begin{array}{rl} \mathrm{and}\; & \frac{\epsilon_{p}v_{e}}{\epsilon_{a}k_{\text{catROS}}}=[\text{MnSOD}]. \end{array}$$

We assumed that the calculation of MnSOD above is the $Q_{\text{Mn}}$ required for cell maintenance ($Q_{\text{Mn},\text{min}}$ in equation (2.2)). In the absence of additional information, we assume identical parameter values for all three PFTs in our model. We test how variations in the efficiency of MnSOD and the reliance on Mn as the sole SOD cofactor in experiments described below. All parameters used for this formulation are in table S1 in the electronic supplementary material.

### (b) Model experiments and metrics

We conducted a set of experiments to quantify how Fe and Mn deficiency develops both spatially and temporally in the Southern Ocean across different PFTs under different phytoplankton traits assumptions. In the following, *limitation* refers to when the micronutrient limited growth falls below the realized growth rate due to light and major nutrients nitrogen and phosphorus (as well as silicon for diatoms, see eqn 4 in [26]) and *co-limitation* is a situation when two limiting nutrients simultaneously affect growth rate [44]. We define *deficiency* as the case when the realized micronutrient quota falls below the required quota, but without any impact on growth [26]. Manganese deficiency is diagnosed from the ratio of Mn quota ($Q_{\text{Mn}}$) to $Q_{\text{Mn},\text{req}}$, i.e the Mn required to cover basal metabolism and photosynthesis (described in §2a). We also calculate the *potential Mn deficiency* based on the Mn quota required when Fe is replete ($Q_{\text{Mn},\text{reqMAX}}$), which is calculated by substituting Fe-limited growth $\mu_{i}^{\text{Fe}}$ with $\mu_{i}$ into equation (2.1):

2.8 $$Q_{\text{Mn},\text{reqMAX}}=\frac{\mu_{i}^{\text{Fe}}}{\text{MnUE}}.$$

*Potential deficiency* is therefore diagnosed from the ratio between $Q_{\text{Mn}}$ and $Q_{\text{Mn},\text{reqMAX}}$ [26]. When the ratio of $Q_{\text{Mn}}$ and the required Mn quota is less than one, then phytoplankton are considered Mn deficient. For Fe deficiency, we use the ratio between excess Fe quota ($Q_{\text{Fe}}-Q_{\text{Fe},\text{req}}$) and $Q_{\text{Opt}}^{\text{Fe}}$.

We conducted eight different experiments divided into two parts, focusing on photo-physiological and nutrient uptake traits, as well as Fe–Mn interactions associated with the production of SOD. These experiments and their short hand names are summarized in table 1. All of the experiments and the control run are initialized from the 250-year spinup of the PISCES-QUOTA with Mn and Zn integrated into the model (similar to PISCES-BYONIC [24,26]), using climatological mean physical field (repeating 1 year physical forcing at 5-day resolution for the entire duration of the simulation), to allow to stabilize biogeochemical tracers using the Hawco *et al*. [26] results as an initialization field. Then the control and experiments are run in parallel for 100 years using the same climatological physical field. We analyse the final year of model output.

**Table 1.** Experiment names and description. The first part of the experiment is similar to that in [26]. Parameter values for each experiments can be found in the electronic supplementary table S1 and [26].

run name	description	Zn and Mn co-limitation (transporter)	QZn hyper-accumulation feedback	high Chl:PSII	low Chl:PSII	iron and Mn co-limitation (MnSOD)
control run		on	off	off	off	off
part I (Mn only)						
antenna large	higher Chl:PSII ratio (see electronic supplementary material, table S1)	on	off	on	off	off
antenna small	lower Chl:PSII ratio (see electronic supplementary material, table S1)	on	off	off	on	off
no Zn–Mn transporter competition	the transporter competition between Zn and Mn is switched off	off	off	off	off	off
Zn hyper-accumulation	downregulation of Zn transport due to internal Zn hyperaccumulation also affects Mn transport	on	on	off	off	off
part II (Mn-Fe interaction)						
MnSOD	explicit representation of MnSOD using default parameter values (see electronic supplementary material, table S1)	on	off	off	off	on
other SODs	assumes phytoplankton meet half their MnSOD requirement by using non-MnSODs %	on	off	off	off	on
antenna large + MnSOD	higher Chl:PSII ratio (see electronic supplementary material, table S1) as well as explicitly represents MnSOD to assess role of lowering Fe limitation	on	off	off	off	on
increase SOD efficiency	increases the maximum turnover rate of SOD	on	off	off	off	on

The photo-physiological and nutrient uptake trait experiments address (1) larger antenna size (Chl:PSII=500 (mol mol^−1^)) and (2) smaller antenna size (Chl:PSII=2000 (mol mol^−1^)). Unlike [26], the modulated antenna size in these experiments also affects the Fe requirement (based on an assumed PSII:PSI ratio and an Fe:PSU, [39]). We also assess traits linked to Mn uptake, using experiments where (3) the competition between bioavailable Mn and Zn for the same transporter is switched off and (4) downregulation of Zn hyperaccumulation affects the Mn uptake (described in eqns 12 and 13 in [26]).

The SOD experiments explore how (1) linking Fe deficiency and Mn deficiency via MnSOD (equations (2.5)–(2.7)) may affect the spatial and temporal distribution of Mn and Fe deficiency. We also explore how different assumptions associated with SOD: where (2) the required MnSOD to combat ROS is halved to explore the reduced reliance on Mn as a cofactor, (3) increasing antenna size (which affects the degree of Fe required and also Mn), and finally increasing (4) the maximum turnover rate ($k_{\text{catROS}}$) of MnSOD as a test of how important variability in enzyme efficiency rates would be.

## 3. Results and discussion

### (a) Extent of Southern Ocean Mn and Fe deficiency

**Figure 1.**
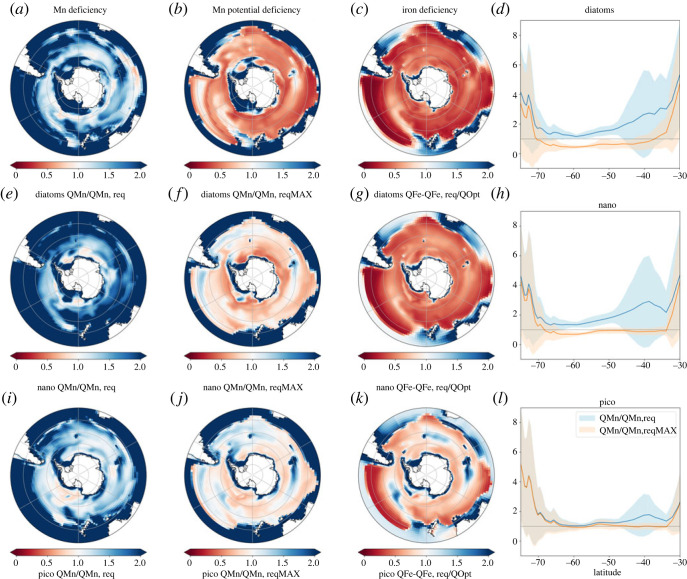
Maximum seasonal Mn deficiency (*a*,*e*,*i*), Mn potential deficiency (*b*,*f*,*j*) and Fe deficiency (*c*,*g*,*k*), as well as zonally averaged seasonal maximum Mn deficiency (blue) and potential Mn deficiency (orange) for diatoms (*a*–*d*), nanophytoplankton (*e*–*h*, nano) and picophytoplankton (*i*–*l*, pico) from the control run. Deficiencies less than 1.0 indicate insufficient resource to support growth and potential limitation.

Overall, the degree of manganese deficiency for all PFTs increases proportionally as $Q_{\text{Mn}}$ declines, MnUE declines and realized growth rates (μ) increase. From the control run, all PFTs have lower $Q_{\text{Mn}}$ within the $60^{\circ }\text{--}70^{\circ }$ S latitude range, but diatoms display the lowest $Q_{\text{Mn}}$ of all PFTs (see the electronic supplementary material, figure S1). In this area, simulated and observed surface dMn is also low (such as central Weddell sea and between $60^{\circ }\text{and }70^{\circ }$ S in the Atlantic sector [21]) and dZn is high [16,20], meaning that phytoplankton cannot maximize their Mn uptake due to both reduced Mn availability and the antagonistic relationship between Zn and Mn [45]. Consequently, all PFTs are actually Mn deficient during the growing season (figure 1*a*,*e*,*i*). In the Indian Ocean sector, around $40^{\circ }$ S, growth rates of all PFTs are relatively high (see the electronic supplementary, figure S1, last panel) due to greater Fe concentrations, and $Q_{\text{Mn}}$ tends to be low, especially for diatoms, driving greater Mn deficiency (figure 1*a*). Additionally, southeast of New Zealand between $40^{\circ }\text{ and }50^{\circ }$ S, high growth rates coincide with low MnUEs, resulting in noticeable Mn deficiency across all PFTs (figure 1*a*,*e*,*i*).

Since the Southern Ocean is strongly Fe deficient (figure 1*c*,*g*,*k*, especially for diatoms), when Fe supply increases, large areas of the Southern Ocean would become Mn deficient (figure 1*b*,*f*,*j*), due to the increased growth rates. Diagnosed by the potential Mn deficiency metric, greater Fe supply would drive Mn deficiency for diatoms, then nanophytoplankton and then picophytoplankton (figure 1*b*,*f*,*j*), suggesting diatoms may be especially vulnerable to the emergence of Mn deficiency under increased Fe supply (by either natural or anthropogenic means). South of $55^{\circ }$ S, picophytoplankton show little difference between Mn deficiency and Mn potential deficiency, indicating they largely remain Mn replete even under if Fe supply increases markedly (figure 1*l*). Mn does not become potentially deficient close to the Antarctic coast and downstream of sub-Antarctic islands, which are regions where combined sedimentary sources of both Mn and Fe are predominant [46,47].

### (b) Traits driving Mn and Fe stress

**Figure 2.**
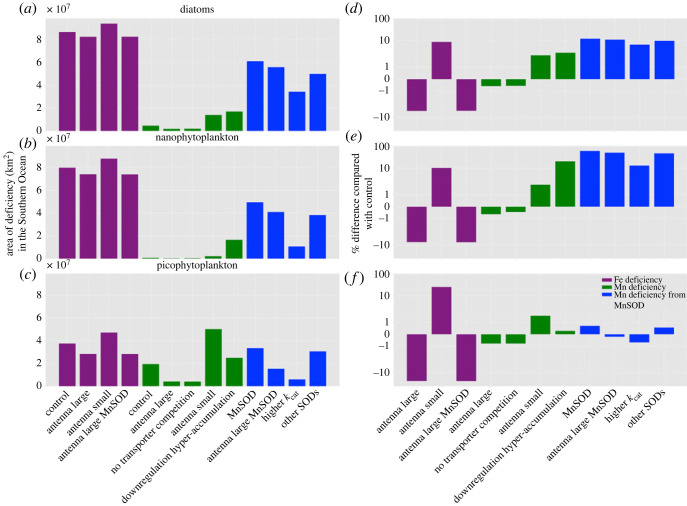
Bar chart showing the extent of Fe and Mn deficiency for diatoms, nanophytoplankton and picophytoplankton from different experiments in ${\mathrm{km}}^{2}$ (*a*–*c*) and the percentage difference between the experiments and the control model (*d*–*f*). Area of deficiency is calculated below $30^{\circ }$ S. The deficiency associated with Fe is shown in purple, Mn deficiency produced from photo-physiology and nutrient uptake is shown in dark green, and from Mn-Fe interactions around oxidative stress in dark blue.

We use the results of our set of trait experiments to explore how the emergence of Fe and Mn stress across the different PFTs. To do so, we delineate the region that is deficient for either Fe or Mn (i.e. $Q/Q_{\text{req}}<1.0$) and examine how its areal footprint changes in response to different trait assumptions. We calculate the area of Mn or Fe stress from the most deficient month, and how these change when different traits are applied. This analysis does not make any distinction of the degree of deficiency, as it focuses solely on areas where Mn and/or Fe is deficient ($Q/Q_{\text{req}}<1.0$). Due to the scarcity of Fe in the Southern Ocean, the extent of Fe stress in diatoms and nanophytoplankton covers much of the Southern Ocean, with diatoms having the largest extent, followed by nano and picophytopankton (figure 1*c*,*g*,*k* and purple bars in figure 2). Laboratory studies have shown that Fe-stressed diatoms increase their Chl:PSII ratio [17,48], with the enlarged antenna size increasing iron use efficiency (and thereby reducing $Q_{\text{Fe},\text{req}}$). Conversely, any reduction in antenna size leads to an increased Fe deficiency by raising $Q_{\text{Fe},\text{req}}$ and reduces light capture [49]. The impact of traits associated with antenna size can affect the extent of Fe stress (purple bars in figure 2), with the largest change shown in picophytoplankton (approx. 12%) as shown in (figure 2*f*). The change in Fe deficiency occurs during the period of Fe stress onset, i.e. in September, October and January for diatoms, nanophytoplankton and picophytoplankton, respectively (see the electronic supplementary material, figure S4). Turning to specific locations, iron deficiency is most sensitive to antenna size traits in the subantarctic region and the $60\text{--}70^{\circ }$ S latitude band at the start of the growing season, but at higher latitudes as Fe becomes more deficient in summer (see the electronic supplementary material, figure S3).

The extent of Mn deficiency shows a strong sensitivity to photophysiological traits. As antenna size increases (higher Chl:PSII), $Q_{\text{Mn},\text{req}}$ declines (see equation (2.2)), and therefore reduces the extent of Mn deficiency, as shown by light green bars in figure 2*d*–*f*. On the contrary, when the antenna is reduced (and by proxy reducing Chl:PSII ratios) the footprint of Mn deficiency increases. Since picophytoplankton have the highest growth rates and tend to have the largest simulated Chl/C ratio (see the electronic supplementary material, figure S1), this PFT displays the largest reduction in their Mn deficiency footprint when the antenna size is increased, followed by nanophytoplankton and then diatoms (figure 2). If the PSII/PSI ratio were to also change alongside antenna size regulation then the parallel change in Fe and Mn stress seen here would become decoupled due to the divergence in the impact on Fe and Mn requirements [26]. Modifications to Mn deficiency due to changing antenna occurs most notably in the subantarctic region within the Indian sector of the Southern Ocean for diatoms and picophytoplankton and southeast of New Zealand for all PFTs, and moves poleward throughout the growing season (electronic supplementary material, figures S3 and S4).

Assumptions regarding the interaction between Mn and Zn around phytoplankton Mn uptake can be important in the Antarctic zone south of the Polar Front. Intuitively, eliminating the competition between Mn and Zn for transporters reduces the extent of Mn deficiency. Since diatoms have the lowest $Q_{\text{Mn}}$, the Zn–Mn transporter competition trait affects diatoms the most (light green bars in figure 2*d*), followed by nanophytoplankton and then picophytoplankton. The extent of Mn deficiency decreases south of the Polar Front closer to Antarctica (electronic supplementary material, figure S4), where dZn concentrations are high and Mn concentrations are also low [16,20]. A study in [50] shows that transporter downregulation occurs when both $Q_{\text{Mn}}$ and $Q_{\text{Zn}}$ are high, to lower the overall uptake of Zn and Mn. In the control model, only downregulation of the Zn uptake occurs, and when this trait is included Mn uptake declines as well. Reducing Mn uptake from Zn downregulation increases the extent of Mn deficiency in the high dZn regions south of the Polar Front, most notably in nanophytoplankton (figure 2*e*), where the extent of Mn deficiency is now almost the same as diatoms (figure 2*b*,*c*). By region, the impact of changing antenna size often coincides with regions where Mn deficiency may occur due to Zn–Mn interactions around Mn transport. This overlap starts to occur in November to January in our model, which is when $Q_{\text{Mn}}$ south of $60^{\circ }$ S are low and Zn concentrations are high (orange colour in electronic supplementary material, figure S4).

### (c) Interaction between Mn and Fe via MnSOD

Accounting for Mn requirements associated with SOD in our model leads to increased Mn deficiency, because any Fe-deficient cell now requires more Mn to deal with oxidative stress. Since all PFTs are Fe deficient through much of the Southern Ocean (especially in summer), the extension of Mn deficiency due to the Mn requirement in SOD can be widespread and often overlaps with Fe deficiency (dark green bars in figure 2). Since diatoms are the most Fe deficient compared with other PFTs, they are also the most vulnerable to an extended Mn stress footprint due to the Mn associated with SOD, with the picophytoplankton much less sensitive than diatoms as picophytoplankton are the least Fe deficient. Seasonal and regional changes in Mn deficiency due to MnSOD can be seen in the electronic supplementary material, figure S5. Of course, the extent of Mn deficiency due SOD is regulated by a set of traits associated with the adaptive strategy used to deal with oxidative stress. Such traits include a larger antenna (to lower Fe limitation), greater $k_{\text{cat}}$ (that reduces the amount of SOD needed) and replacing MnSOD with other forms of SOD that use alternative cofactors (table 1).

When MnSOD is accounted for, increasing the antenna size will lead to reduced Fe deficiency and hence reduced SOD requirement. When we include this trait in our SOD experiments, there is a reduction in the Mn-deficient area, most notably for picophytoplankton (figure 2*d*–*f* in purple). Increasing the catalytic efficiency results in higher consumption of ROS per MnSOD and hence also reduces the extent of Mn deficiency, with the greater reductions than those seen for a greater antenna size (figure 2*a*–*c* in light green). For picophytoplankton, the extent of Mn deficiency from imposing higher MnSOD catalytic efficiency is lower than the control model (figure 2*f*), highlighting a strong sensitivity to this trait, especially in PFTs that are the least Fe deficient. If we assumed a greater reliance on non-MnSODs (such as Fe or copper, shaded green in the electronic supplementary material, figure S5), there is only a small reduction in the Mn-deficient area (figure 2). However, between December and January for diatoms and nanophytoplankton, the area of Mn deficiency due to MnSOD can be strong modulated by reducing the reliance on MnSODs (electronic supplementary material, figure S5).

### (d) Implications for net primary production and the biological carbon pump

**Figure 3.**
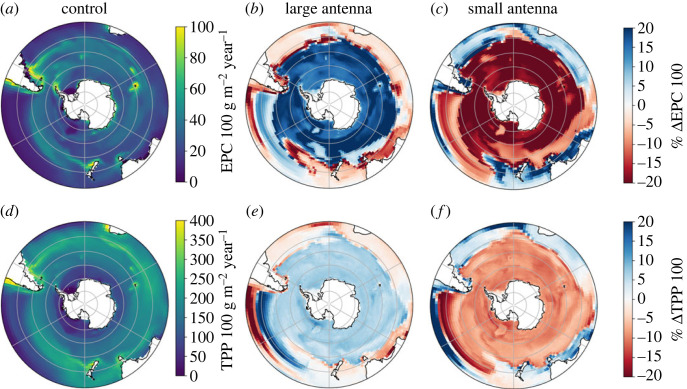
Carbon export at 100 m (EPC 100, (*a*)) and total primary production (TPP 100, (*d*)) from the control run integrated over the upper 100 m in the Southern Ocean integrated over 1 year, compared with experiments with large antenna (*b*,*e*), and small antenna (*c*,*f*). The top and bottom row show the difference between EPC and TPP, respectively. Δ is the percentage change (experiment—control run).

Assumptions regarding the suite of physiological traits also affect regional rates of NPP and the biological carbon pump (BCP as estimated by the carbon export across 100 m). The strongest effect is seen in response to changes in antenna size, either greater or lower than the average value and fixed Fe per PSU assumed in our model. Alterations in antenna size and the Fe and Mn required in photosynthesis lead to changes in the BCP of greater than  20% south of $40^{\circ }$ S (figure 3*b*,*c*) or up to 20% for NPP in the East Pacific sector of the subpolar region (figure 3*e*,*f*). This suggests that if Southern Ocean phytoplankton are parameterized with unique adaptations to low Fe via their photosynthetic antenna size and Fe requirements of a photosynthetic unit [17] a strong sensitivity to the regional carbon cycle would result. Accounting for the Mn required to deal with oxidative stress also affects BCP and NPP, although not as much as changing antenna size. Changes in NPP and BCP can be decoupled due to changes in the relative contribution of diatoms and picophytoplankton in this experiment (electronic supplementary material, figure S6), since these PFTs contribute the most to NPP (41.7% and 34.0% in the Southern Ocean, respectively, from the MnSOD experiment). This indicates that the taxa or species-specific traits associated with oxidative stress management, such as the reliance on MnSOD or the enzymatic efficiency of SOD, will have implications for NPP and the biological carbon pump under changing Fe supply. In this case, the sensitivity to Mn limitation and the C cycle impacts may be amplified or dampened, depending on the physiological strategy employed by Southern Ocean phytoplankton. This will operate alongside environmentally driven changes in the physical supply of Fe and Mn to different Southern Ocean regions.

### (e) Model limitations and future directions

The Southern Ocean is a unique environment and is rich in diatom species in particular [51]. Laboratory work has shown that Southern Ocean groups have specialist traits and display different acclimatory responses relative to those seen in temperate species. For instance, Southern Ocean phytoplankton groups have evolved to exploit different iron sources [52] and dynamically change their PSII/PSI ratio and photosynthetic antenna size to lower their $Q_{\text{Fe},\text{req}}$ under Fe-limited conditions [17,48]. Including Southern Ocean-specific parameterizations can improve aspects of model performance [53], but previous efforts have relied on abrupt latitude dependent shifts in model parameterization. Even though our global model represents three PFTs, we have not made distinctions in the photophysiology or nutrient requirements of Southern Ocean groups and we ignore Southern Ocean-specific phytoplankton (such as *Phaeocystis antarctica*), as is common for all global ocean biogeochemical models. Our sensitivity experiments demonstrate how locally adapted traits may play a role in regulating nice differentiation in the region by governing the extent of Fe and Mn deficiency. Future work that refines the photophysiological and oxidative stress traits, such as quantifying the efficacy of ROS consumption for different PFTs, will prove critical in better constraining regional patterns of resource limitation. In that context, expanding the extent of experimental work to cover broader regimes and the responses of different phytoplankton groups (e.g. [7,23]) would be essential.

In this work, we explored how changes in a suite of physiological traits affect the extent of Fe and Mn deficiency, either within or across phytoplankton groups. Ultimately, addressing the influence of changing Fe and Mn limitation in a changing climate is a pressing need. Currently, CMIP6 models project future increases in NPP with relatively high confidence, but we do not know how accounting for the emergence of Mn limitation may affect these results, especially at regional scales and for specific phytoplankton groups. Further work, to quantify how the different sources, sinks and transport of Mn and Fe may be affected by a changing climate, would be important. This may be especially important given that changes in atmospheric deposition of Fe and Mn are changing regionally (e.g. [54]) alongside broader modifications to ocean circulation and mixing (e.g. [55]).

Our model results point to oxidative stress and photophysiology as a potentially critical interaction between Mn and Fe. However, our approach to representing MnSOD in our global model was relatively simplistic. In the proteomic model based on the polar diatom *F. cylindrus* [30], a penalty of a reduced protein synthesis rate is applied if the Mn quota is not met to account for the oxidative damage. This is supported by laboratory study of Southern Ocean diatoms [19], where adding Mn in Fe-starved diatoms would increase the growth rate, but the photosynthetic carbon fixation rate is not restored. In order to better understand Fe–Mn co-limitation at the Southern Ocean, a chain-model-like approach may be needed to capture these trade-offs and co-limitation processes [56]. For example, it is important to account for dynamic PSII/PSI ratios, which set the Fe content of a photosynthetic unit (PSU, three Fe atoms in PSII compared with 12 Fe atoms in PSI [15]) and changes in the antenna size (i.e. modulating Chl PSU-1), in Southern Ocean diatoms, as this can also affect Fe and Mn stress. Increasing PSII/PSI ratios would lower Fe demands, but it would make PSII less efficient [57] and require a further four Mn atoms for each PSII [26]. By employing a more realistic representation of Mn-Fe co-limitation, based on the studies above, it is possible that Mn stress can alter greatly our estimates of BCP and NPP.

## 4. Conclusion

We conducted a set of trait-based experiments with a state-of-the-art ocean biogeochemical model, including three PFTs and explicit consideration of Mn limitation and deficiency. We found that phytoplankton can be Mn deficient and this can impact the NPP and the biological carbon pump in the subantarctic Southern Ocean. Without the inclusion of Fe–Mn interactions around oxidative stress, Mn deficiency is not as widespread and is mostly concentrated near Antarctica, southeast of New Zealand and the Indian Ocean, due to low Mn quota, caused by high surface dZn and high growth rates. The footprint of Mn deficiency is exacerbated when Fe–Mn co-limitation around oxidative stress is included in the model, especially for diatoms and nanophytoplankton. Alternative resource uptake traits and higher maximum turnover rates of MnSOD would reduce the extent of Mn deficiency. Photophysiological adaptation, such as changing antenna size, can be beneficial in combatting both Mn and Fe stress in Southern Ocean phytoplankton, and has overall greatest impact on NPP and the biological carbon pump because it impacts the degree of Fe limitation. Further observation, experimental and modelling work are needed to develop further the parameterization of interactions between Fe and Mn in the photosystems and around oxidative stress and address how Fe–Mn limitation regulates the response of Southern Ocean NPP and the biological carbon pump to a changing climate. New insights into the range of physiological traits deployed by different groups across different Southern Ocean regimes from a suite of tools, including genomics, bio-optics and manipulation experiments, would be necessary to improve regional estimates of the carbon cycle impacts.

## Data Availability

Model outputs are available from: https://doi.org/10.5281/zenodo.7788243 [58]. Supplementary material is available in [59].
